# John Perfect Shares Insights on Infectious Diseases, Antifungal Therapy, and Drug Resistance

**DOI:** 10.20411/pai.v10i2.886

**Published:** 2025-09-05

**Authors:** Mahmoud Ghannoum, Robert A. Bonomo

**Affiliations:** 1 Department of Dermatology, Case Western Reserve University School of Medicine; University Hospitals Cleveland Medical Center, Cleveland Ohio; 2 Medical Service, VA Northeast Ohio Health Care System Louis Stokes Cleveland Department of Veterans Affairs Medical Center, Cleveland, Ohio; 3 Department of Biochemistry, Case Western Reserve University, Cleveland, Ohio; 4 Department of Medicine, Case Western Reserve University, Cleveland, Ohio; 5 Geriatric Research, Education and Clinical Center, Louis Stokes Cleveland Department of Veterans Affairs Medical Center, Cleveland, Ohio; 6 Departments of Pharmacology, Molecular Biology & Microbiology, and Proteomics & Bioinformatics, Case Western Reserve University, Cleveland, Ohio; 7 CWRU-Cleveland VAMC Center for Antimicrobial Resistance and Epidemiology (Case VA CARES), Cleveland, Ohio

**Keywords:** Cryptococcus, HIV/AIDS, Antifungal Therapies, Drug Resistance

## Abstract

In this engaging interview, Dr. John Perfect reflects on his distinguished career in infectious diseases, focusing on his early scientific interests, his pivotal experiences during the emergence of HIV/AIDS, and his extensive research on fungal pathogens, particularly *Cryptococcus*. Dr. Perfect discusses the evolution of antifungal therapies, the challenges of drug resistance, the promise of molecular diagnostics and immunotherapies, and the complexities faced in clinical research. He emphasizes the importance of mentorship and optimism for future generations of scientists, concluding with a message of hope and encouragement for ongoing innovation and resilience in medical science.

## ROBERT BONOMO, MD

It is our pleasure to interview today, Professor John Perfect. Professor Perfect is the James B. Duke Professor of Medicine at Duke University School of Medicine. He is also the Chief of the Division of Infectious Diseases and a Professor of Molecular Genetics and Microbiology at the Duke University School of Medicine. Over the years, Dr. Perfect has accumulated many accolades and awards.

In addition to his current positions, he is the Director of the Duke University Mycology Interdisciplinary Research Unit. He's a Fellow of the Infectious Diseases Society of America, and a Fellow of both the Association for the Advancement of Science and the Academy of the American Microbiology Society. He is a member of the American Association for Physicians, which is a distinguished society for physician scientists. He's also past president of both the Mycoses Study Group Education and Research Symposium and the International Society for Human and Animal Mycology. Dr. Perfect received his undergraduate degree in biology from Wittenberg University and his medical degree from the Medical College of Ohio in Toledo.

After completing his residency at the University of Michigan in Ann Arbor, he completed an infectious disease fellowship at Duke University in Durham, North Carolina, where he has stayed and flourished as an internationally recognized physician scientist. In addition, he is the principal investigator for multiple National Institutes of Health (NIH)-sponsored interdisciplinary antifungal drug programs and conducts research on various aspects of molecular pathogenesis in fungi, particularly *Cryptococcus*.

John has more than 600 peer-reviewed publications, journal articles, book chapters, books, and reviews. He's a member of the Guidelines for the Prevention and Treatment of Opportunistic Infections, which is a collaboration between the NIH, Centers for Disease Control and Prevention (CDC), the HIV Medicine Association (HIVMA), and the Infectious Diseases Society of America (IDSA). He has also been an excellent teacher, earning Duke University's Educator of the Year Award, and has received many mycology awards, including the Littman Award, the Rhoda Benham Award, and the Lucille Georg Award.

So, John, tell us about your childhood. What were the roots of your success?

## JOHN PERFECT, MD

Well, thank you very much. I will add some personal statements to that introduction. I am from Ohio. I grew up in Johnstown, Ohio, which is outside of Columbus, Ohio. I went to Wittenberg, and eventually to the Medical College of Ohio. And believe it or not, I then went up north to do my training in medicine at the University of Michigan.

So, how did I start? I was always interested in science and in simple concepts that help people. I tell that to anyone who asks why I do this. If you go into medicine, I think, you have a desire to help people — very simply. I was an individual who enjoyed making a diagnosis, providing a treatment, and curing the patient. Infectious disease is an area that has this characteristic. You would discover something, design something to understand it, and then create something to treat it.

However, things change. Around the early 1980s, there was a disease process in immunocom promised young people. It turned out to be HIV, and it was a problem, because we didn't have a treatment. And there were many nights when I saw young people die from HIV.

It's been just dramatic to see the progress that's been made in a disease process where there was no treatment to one that is now a chronic disease process to manage. During the AIDS epidemic, I had to care for patients without treatments. This was a dramatic change for me. And it was traumatic to see young people die. During that time, one of the most important aspects of patient care I've ever experienced was taking care of patients when I had no cure to offer.

Another thing that was sad was seeing how other people treated some of these patients, the modern-day lepers, so to speak. I was also saddened when my colleagues in other specialties didn't want their patients to be in the same room as mine.

Now, HIV is a chronic disease process. It's the diabetes, so to speak, of infectious diseases, which was made possible by the people who had to care for these patients in the early stages of this epidemic. It was their ability to develop treatments and get these patients into a chronic disease process. My only hope is for a vaccine, and I don't know if one will be developed in my lifetime.

## MAHMOUD GHANNOUM, PHD

What you said is what keeps us in this business: you want to help people. Sometimes people ask me if I'm going to retire, and I tell them I'm still enjoying myself. It is fantastic to hear what keeps you going.

## RB

I think we all went through that. I remember in 1986, I finished my residency, and I went out into private practice, and I saw all these young men present with oral thrush. It was heartbreaking to be part of healthcare at that time, when you knew that this was the first sign of AIDS and that they would be admitted to the hospital with what was then called pneumocystis carinii pneumonia, PCP. It was a rough go. It would break your heart to see these young men move on. And since then, the HIV epidemic, if you will, changed.

## MG

As a clinician, you have recent experiences with the epidemiological trends in candidemia. How has candidemia changed over the years?

## JP

I don't know if much has changed. I was looking at this in our own hospital, where the incidence of candidemia has been very high for 20 to 30 years, largely due to the broad-spectrum antibiotics that we give and the tubes that we use, which are hallmarks of modern medicine. But we recently saw a decline in cases of candidemia. We typically see 120 to 140 cases per year. Why did it decline? I suspect it was due to a shortage of blood culture vials. We are a hospital that uses 30-mL vials, and we had to temporarily switch to 20-mL vials. Recently, we've gone back to our 30-mL vials, and there is plenty of candidemia out there again. I suspect that volume makes a difference in detection rates. The high incidence seems consistent over time.

Many times, patients with candidemia are pretty sick due to other disease processes, and we're seeing them at the end of life. So, the terminal mortality rates, around 20% to 40%, reflect this severity.

A sad part of this is that we have a hard time doing candidemia studies in the United States because of how sick these patients are and how complex the end-of-life stages are for many patients. We will screen 50 patients to enroll 1 patient in a study, because the patients have numerous underlying diseases.

## RB

What do you see as the role of these molecular-based diagnostic methods currently being developed for non-culture-based testing, which are obviously based on molecular signals that can be found in blood samples? What do you think their role will be in the landscape of diagnosing can-didemia? In other words, do you think we will encounter many false positives or false negatives? What role do you see these rapid, molecular diagnostic tests playing in this scenario?

## JP

That's a good question. I believe that over time, they will become players because they will eventually be streamlined and validated. We will all need to deal with it because, again, all our systems have been based on cultures; they are not based on DNA, which is a lot of what these things are: DNA and RNA. I think there will be some evolution to these things. But I do see, probably over time, they will eventually be integrated into clinical practice. I see them being bigger players. There will be some clinical evolution.

## MG

A number of companies are reaching out to me to develop quick diagnostics that differ from PCR, so I agree. This is going to happen. And the most important thing is the validation. I'm glad these companies are now starting the first step towards validating these approaches. So yes, we are going to see more of that.

## JP

If I could share my recent personal experience with this kind of testing. My daughter, who's a physician here at Duke, had pulmonary disease — coughing, fever, various things, and it went on for weeks. She received antibiotics. When she went to her primary care doctor, they performed a rapid test for 15 different pathogens in her airway. It turned out, after all these antibiotics that didn't work, she had mycoplasma. After we made the diagnosis through this testing, she received the right antibiotic and improved quickly. So, what I want to say is that, over time, the ability to do these things will be here, and I'm actually in favor of that. There are many things out there, and testing is continually improving.

## MG

You've been working with *Cryptococcus* for a long time, so I want to ask you what is new?

## JP

It all began on Valentine's Day, February 14th, 1978, at 11 PM. If you look at one of my earlier papers, you'll find a note I wrote from when I was a fellow, describing a patient I saw with cryptococcal meningitis [1]. That night, I examined an isolate under India ink, and that is the isolate I worked with for 48 years. I named it H99 after listing it in my boss's book as the 99th organism in the human isolate collection. That's how it became H99. I share this background because it was a fluke that I happened to be there—and I have since dedicated 48 years to studying that single organism, which we now know is actually more than one organism.

As much as I kind of hate and love H99, that organism gave us a lot of tools. What we are trying to do now is sequence a lot of strains. H99 is one strain. These organisms evolve and adapt; so we should never assume that one organism is telling us everything. I've now collected thousands of strains of organisms to better understand their structures. What I anticipate in the next 15 to 30 years is that we'll be able to see how strains evolve on both macro and micro levels.

I'm still as excited about medicine and research as I was in 1978 when I first saw H99 under a microscope. So, despite today's challenges, I urge young scientists not to give up. The system is too good. The change from what we could do when I started to what we can do today is dramatic. The future is absolutely bright when it comes to science.

And you're asking, are there new drugs or new compounds? I'm a little biased right now, because I'm working on a compound that I think is the best cryptococcal drug that I've ever seen. If it works in humans like it does in the animals, we could someday potentially treat cryptococcal meningitis for 2 weeks and call it a day. But I've learned to be cautious with aggressive treatments. Understanding the host's response is crucial. So, another concept I want to leave young people with is that it's the Goldilocks paradigm day in and day out when it comes to immunity. Equilibrium is essential in managing infections because immune responses can escalate or overshoot.

Ultimately, the art of medicine remains very important because our diagnostic tools are still evolving, and maintaining the right immune balance is key.

## RB

Each of my grants is based on a clinical isolate I discovered in a patient. I remember December 28th, 1984. It was approaching New Year's Eve, and we had a patient at our hospital who had an ESBL bacteremia. I called Dr. David Shlaes to ask how we treat this. And I went on to keep that isolate. I was a fellow under Dave when I went back to do my ID fellowship, and we got the crystal structure of that enzyme for the first time at 0.9 angstrom resolution. I don't know how that happened, but these serendipitous things are funny.

You've been involved in the development and testing of multiple antifungals that have been introduced into the clinic and in various capacities. What were the lessons you learned with each new introduction of a novel drug? What were the important lessons as a physician-scientist and as a clinician? What did you learn from the introduction of X antifungal? How is it applied to another antifungal that you had the opportunity to test? And what mistakes do you think were made in the evolution of this field that would be interesting to know?

## JP

When I started, there was a drug called amphotericin, and I think it appropriately got its name as amphi-terrible for a reason. I have seen amphotericin from its days as a pretty toxic drug. I haven't used amphotericin deoxycholate in 5 or 10 years. It's gone. So, we evolved a pretty toxic, potent, broad-spectrum antifungal to one that's more usable, particularly in medicine today.

Second, I also found the growth of the azole compounds to be extraordinary. They went from nail infections, yeast infections, vaginal candidiasis, to overwhelming candidemia. You see these things improve over time, with long half-lives and a little broader spectrum. And the progress from when they first started to where they're at today is remarkable. I think they're a great story.

The echinocandins are another great story. They were the penicillin, so to speak, of fungi. We knew that this had to be attacked with fungicidal-type drugs. And the ability to bring them into the clinics now is impressive; in fact, they're the standard first-line treatment for candidemia. I think it's remarkable what they've achieved.

So, without going too much further, each one of those classes has had a story around it, and each one has progressed very well. Do I think they're perfect? No. And I'll tell you why I think we need to continue studying them.

Not only do they sometimes lack the broadness that they need, but the other thing that is sad to me about all three of these classes, and it may have to do with drug development and various other things, is that I think we've made a mistake. These fungal infections need to be treated for long periods of time, weeks to months. When we do these studies, we shouldn't be thinking about that. Just as I mentioned the potential of maybe someday treating cryptococcal meningitis for 2 weeks, we need better fungicidal drugs that get in and get out. We should not be keeping people on these drugs for weeks and months like we do right now. Don't get me wrong. I'm not changing that game. I'm simply saying that drug development should focus on potency and the ability to diagnose, treat, and then withdraw treatment rapidly, so we avoid conflicts or complications around the underlying disease. Remember, when you're dealing with fungi, you're often dealing with a very serious underlying disease, and you want to avoid complications like amphotericin B and azole drug interactions.

That's a long answer to a very interesting question, but if you look at these three classes, each has a notable history. I hope and pray that newer classes and more potent drugs will emerge, allowing us to treat patients even faster. That may not make it successful for the pharmaceutical company, per se, but I will bet it will make it successful for the management of the patients.

## MG

I can't leave this interview without asking you the question. What about anti-fungal resistance? Should we be more concerned compared to antibacterial resistance?

## JP

I've published papers on this, so you have an idea about my thoughts around antifungal drug resistance [2–5].

It's a multi-factorial layered-type situation. One thing I would like to say from the fungal standpoint is, unlike with bacteria, there's no plasmid-mediated, transposon-mediated drug resistance.

I don't think we're in the same class as bacterial stuff. Not to say that you can't develop resistance on these drugs, but I don't think it's quite the same. What I would say is that there is some drug resistance. Look at what's happened with *Candida auris*. There's a moderate amount of resistance with it. That depends on where you're at. I hate to say it, and I'll knock on wood. I watch how so many people are now overwhelmed with *C. auris* infections, and in some cases, there is drug resistance. That's still very specific for certain locations. For instance, we at Duke see very few cases, and the cases we've seen have been with fairly antifungal susceptible isolates. So, it's a variable situation with drug resistance depending on where you're at. But I think the ability for drug resistance to develop and occur is concerning.

The last aspect I see regarding drug resistance in fungal infections isn't so much about the mechanism, but what I call clinical resistance — when the patient fails treatment. This may be due to toxicities or other underlying conditions. That's what I refer to as clinical resistance. Fungal infections often sit right in the middle of this because they are linked to these very active underlying diseases. And those things will require insights from many different angles when it comes to fungal infections. There's plenty of resistance out there. There will continue to be, but in my view, the biggest component of resistance is clinical resistance. There are just a lot of factors underlying disease toxicity.

## MG

I know we are focusing on more systemic infections, but more recently, I'm sure you both heard about the *Trichophyton indotineae,* which started in India [6]. The CDC is interested in doing a survey to see to what extent we are seeing this in the United States. We published a paper with the CDC showing that one patient who was traveling from Southeast Asia acquired it, and it was sexually transmitted [7]. So, it's becoming increasingly complicated, beyond just having resistance to toenail infections, for example. I think it would be wise for us to consider this more often, because I completely agree with you on the comparison between bacterial and fungal resistance. This issue with antifungal resistance is increasing now in both systemic and cutaneous cases.

## JP

You're absolutely right. I don't want to shortchange that. Particularly regarding dermatophytes and similar conditions, you can imagine that various treatments are available over the counter. What you see now isn't going to change, and we're likely to see more drug-resistant isolates emerge. Even in the fungal field, we must be cautious in our use of drugs, particularly systemic medications, as many of our patients have serious underlying diseases or compromised immunity, resulting in a high burden of organisms. This is still a fertile area for drug resistance, whether it's on the skin or in immunocompromised hosts. We do need to be aware of those things.

I also want to emphasize that when it comes to bacteria, Robert will face greater challenges, and it gives me chills to think about the spread of resistance through transposons and plasmids. I hate to say it, but I'm on the fungal side. I'm a big cheerleader for more antifungals. As a clinician, I am concerned with the lack of progress in developing new drugs and new mechanisms to combat bacteria. We've discussed this for many years, but I still don't see significant advancements, and that continues to bother and scare me.

## RB

We've talked about anti-fungal therapy. We've discussed anti-fungal diagnostics, molecular methods, and the evolution of these processes, but do you think we will ever have immunologic methods to treat fungal diseases? In other words, will we be giving an antifungal and a monoclonal antibody? Will we be giving an antifungal and a cytokine? Will we give a biological modifier and an antifungal?

## JP

When we talk about these things, we talk about two things: the host and the parasite, or the host and the fungus. We spend a lot of time on the fungus and direct antifungals, among other things. But you're absolutely right that we need to build up the host. Clinical resistance is important.

The first area that is interesting is gamma interferon. Several large studies have shown quite positive outcomes for treatment in cryptococcal meningitis [8, 9]. When you look at the pathophysiology, you can see how adding gamma interferon to this condition might improve the killing of the fungus. My only concern is that we have monoclonal antibodies and cytokines, but I'm not sure we're quite there yet in measuring these patients effectively. In other words, we need to know where they stand in their immune response.

We introduce these powerful agents and can go either way—perhaps too quickly or without enough caution. That worries me a bit. I hope that as we develop these therapies—monoclonal antibodies, interferons, and others—we also develop monitoring systems to understand each patient's immune status better. Because too much or too little response isn't beneficial. We need to get it just right. I believe we must improve in that regard as we evaluate new treatments. It concerns me somewhat to introduce all these immune modulators, risking overshooting.

I'm all for continuing research in this area. I do believe there's real potential because, the truth of the matter is, if you don't improve the host, all these antibiotics and antifungals will only be mediocre at best.

## MG

I looked at the article where you talked about cryptococcal nutrient acquisition and dining on the host [10]. Again, it brings those into the picture. Can you tell us about this?

## JP

One of the things we study when we examine the organism is what it does and how it survives. What are its tools? Think about this a little bit. In cryptococcus, it's got to live in that host, and there are a few million fungal species, and they don't dine on us very much. They just can't deal with us. Some of that is related to temperature. We're kind of hot, and most of these fungi can't survive in that heat. But they also have to be able to grow or replicate in some way, shape, or form.

Remember, when you think about fungi, you're already talking about maybe 300 species that have caused disease in humans. They had to live in a hot environment or at least survive at a hot temperature. They must have access to nutrients and the ability to grow, replicate, or survive.

I think some of the fundamental things that we've been studying are how they deal with carbon, how they deal with nitrogen. I think the next generation of biologists, pathologists, and clinicians should consider how these organisms can thrive in an environment they may not particularly enjoy, but in which they can still make a significant impact. Those types of areas are where drugs or vaccines can still be very attractive, because some of these organisms have had to develop tools to survive in a pretty inhospitable environment, which is the human host.

Without going into the details of dining on the host, for the people thinking about this, pay attention to what that is and what they've had to do. They've had to develop tools to be able to live in the human body. Unlike viruses, which are required to live in living hosts, fungi can live in various environments. So, these are very attractive areas to continue to study.

## RB

All three of us have been in the academic world for a long time, and all of us have had trainees that have rotated through our labs. We've had the privilege to bring people along in their academic careers and mentor them. Share with us your feelings or your concerns about the future. Are we beginning to lose scientists? How important are the educational efforts we have? And how could we best preserve them?

## JP

Robert ends with one of the toughest questions and one of the most important questions. What I would like to leave with is that, although things may evolve, we need to be much more optimistic. We have incredible tools. I know there are issues around funding. There are issues surrounding the evolution of health care. But for the young people getting into science, these are snapshots of what we're dealing with today. They will be figured out. The important thing is that the scientific principles and the structures that have been developed are sound. I have never been more excited. Science is evolving, and it might cause some wobbling for a little while. But for anyone interested in medicine, science, or related fields, there's never been a better time than now. Even though we may face challenges, these will be temporary. The principles are there. They're being taught. There are resources, even if they're shifting. There's excitement, and there should be excitement, because we can make a lot of progress. We really can. The fundamentals are strong, and with hard work and enthusiasm, it will be rewarding.

## RB

On behalf of Dr. Michael Lederman, the editor in chief of *Pathogens and Immunity,* and the rest of our editorial board, we want to thank you for this outstanding discussion. We really appreciate your enthusiasm, that we need to press on, and that we are at an exciting time. We've learned so much. We can't let this go.

## JP

That's a great summary. And to both you and Mahmoud, thank you for asking me. Hopefully, I have left with a positive message, because it is a positive message. We need to move forward. We have incredible tools. We have incredible people. And it's all right there. And for the next generation, go for it.

## SUPPLEMENTARY DATA

Supplementary materials are available at the *Pathogens and Immunity* website. Supplementary data may be provided by the authors to benefit the reader. Supplementary data are not copyedited and are the sole responsibility of the authors. Questions or comments related to supplementary materials should be addressed to the corresponding author.
